# Integration of Context Awareness in Smart Service Provision System Based on Wireless Sensor Networks for Sustainable Cargo Transportation

**DOI:** 10.3390/s21155140

**Published:** 2021-07-29

**Authors:** Dalė Dzemydienė, Aurelija Burinskienė

**Affiliations:** 1 Department of Business Technologies and Entrepreneurship, Faculty of Business Management, Vilnius Gediminas Technical University, LT-10223 Vilnius, Lithuania; aurelija.burinskiene@vilniustech.lt; 2 Institute of Data Science and Digital Technologies, Faculty of Mathematics and Informatics, Vilnius University, LT-08412 Vilnius, Lithuania

**Keywords:** smart service provision system, context-aware services, wireless sensor networks (WSNs), information communication technologies (ICTs), cargo transportation

## Abstract

Smart service provision systems can assist in the management of cargo transportation. The development of these systems faces a number of issues that relate to the analysis of numerous factors, which are influenced by the properties of such complex and dynamic systems. The aim of this research was the development of an adaptable smart service provision system that is able to recognize a wide spectrum of contextual information, which is obtained from different services and heterogeneous devices of wireless sensor networks (WSNs). To ensure that the smart service provision system can assist with the analysis of specific cases of unforeseen and unwanted situations during the cargo transportation process, the system must have additional adaptability. To address the adequate provision of contextual data, we examined the problems of multi-dimensional definitions of contextual data and the choice of appropriate artificial intelligence (AI) methods for recognition of contextual information. The objectives relate to prioritizing potential service provision by ensuring the optimal quality of data supply channels and avoiding the flooding of wireless communication channels. The proposed methodology is based on methods of smart system architecture development that integrate the identification of context-aware data, conceptual structures of data warehouses, and algorithms for the recognition of transportation situations based on AI methods. Experimental research is outlined to illustrate the algorithmic analysis of the prototype system using an appropriate simulation environment.

## 1. Introduction

Researchers are currently concerned with the problem of integrating smart components into the development of systems that can be applied to the management of cargo transportation processes [1,2]. Important issues have arisen in the development of advanced tools based on innovative information and communication technologies (ICTs), which have the potential to adapt to the heterogeneity of communication infrastructure and the diversity of vehicle automation tools [3,4]. 

To recognize data provided by wireless sensor networks (WSNs), further detailed investigations are necessary for the development of smart service provision systems [5,6]. The development of intelligent transport systems (ITSs) requires new methods, information communication technology (ICT), and possibilities for the adaptation of the heterogeneous infrastructure of WSNs.

The European Union’s (EU) planned digitization activities promise to help in the development of this type of infrastructure [7,8]. The New Digital Agenda for Europe [9] outlines important objectives to be achieved by 2030 that are currently influencing the development of ITSs. These objectives are affecting cargo transportation processes and are related to the goals of the Sustainable Development Agenda 2030 [10]. The new Strategy for ICT development until 2030 [9] notes several significant innovations, i.e., state-of-the-art ambitions, which include a focus on artificial intelligence (AI), cloud computing and block-chain technology via supercomputers and quantum technologies, and the use of the Internet of Things (IoT). The most important obligations and opportunities are formulated as follows:

- Efforts have been concentrated on the development of the Single Digital Gateway (SDG) [11], by which SDG Regulation imposes an obligation on all EU Member States to provide access to information regarding new kinds of public services (which are formulated as 21 procedures), and e-mail services used in seven key life events will become fully digitalized (i.e., the whole service delivery process takes place online and cross-borders).
- Support of efforts for the implementation of the free movement of data and the protection of personal data [9]. Strict obligations for the EU Member States are created by the General Data Protection Regulation and the Open Data Directive [12].
- New approaches to the development of cybersecurity as an objective of the Information Systems Directive [13,14]. Stronger attention to the cybersecurity objective imposes the obligation on the Member States to achieve an appropriate level of preparedness against cyberattacks.

These innovations influence opportunities for the development of infrastructure for intelligent service provision. The most important requirement for the development of ITSs is that these systems do not distract drivers during the transportation process. The transport management system should provide the required services to the users in the right place, at the right time. Furthermore, it should be appropriately adapted to the needs of the users, by making only the interventions that are necessary for the safety of the transportation process. 

The objective of this research was to develop a smart service provision system for cargo transportation management. The developed system aims to meet the requirements of usefulness, adaptability, safety, and performance optimality by creating a working regime that does not flood wireless communication channels. Our objectives are related to the stages of the creation of the smart service delivery system with the functions of monitoring and recognizing contextual information. Context-aware information must respond to the main needs of cargo transportation processes. The platform for the developed smart service provision system is based on the infrastructure of wireless sensor networks (WSNs). The recognition of the obtained information was implemented in the stages of transportation and enabled sustainable cargo transportation management. 

The proposed approach is based on construction methods for the development of a component-based architecture of a smart adaptive service delivery system (IASDS). The results obtained in previous studies of the description of the ICT infrastructure of multi-modal transport management are also relevant to the current work [4,7,8]. It is hoped that the current investigations will assist in the development of autonomous transport infrastructure with extensible heterogeneous communication, and thus enhance the safety of these processes. However, we did not examine cybersecurity issues in the current study because such issues require a different approach and, as such, they are outside the scope of our research.

A formalized understanding of the current and previous developments in transportation processes is necessary to increase the autonomy of user–vehicle interactions. AI-based methods have been applied to the forecasting of possible future situations. The ICT infrastructure includes a variety of new kinds of wireless communication channels and WSNs [5,6,7,8]. The integration of heterogeneous services with platforms based on the geographical information system (GIS) is vital to the online recognition of coordinates of moving objects. The development of smart system structures has supported the integration of data from sensors into the data monitoring processes of moving vehicles and the development of systems for process forecasting based on the assessment of data about the vehicle’s surroundings. In this research, we aimed to concentrate our attention on the problems of restricting unnecessary information, prioritizing the service provision process, and formalizing the limitation of information flows due to excessive data transmissions, with an overall focus on increasing driving safety. 

The structure of this article is as follows: Section 2 is devoted to reviewing research related to investigations of context-aware processes and systems development. An approach (i.e., methodology) for the development of an adaptable smart service provision system, with a focus on the presentation of the component-based architecture of the proposed system, is presented in Section 3. Cases of possible conflicts in the scheduling processes of cargo transportation and possibilities for resolution are described in Section 4. Algorithms used for classification and restriction of contextual information for drivers, based on the background of AI and mathematical methods for decision support, are presented in Section 5. Experimental research possibilities are briefly described in Section 6. The limitations of, and future plans for, our research are presented in the Section 7. A summary and the scientific novelty of this study are presented in the Section 8.

## 2. Review of Related Works and Analysis of Unsolved Problems

The development of new kinds of services for transportation support is related to the development of ITSs and the spread in the implementation of the advanced functions of ICT [4,15]. New kinds of wireless sensor systems equipped at the roadside and in vehicles have been applied to the management and control of transport processes [16,17,18,19,20,21]. However, an adaptable interaction between transport processes and the infrastructure of roadside units (RSUs) requires further research, with particular attention paid to smart and autonomously working subsystems. 

Vehicle ad-hoc networks (VANETs) [20] can be deployed in a number of ways, some of which are supported on methods of long-distance communication, based on mobile protocols such as 4G, 5G, or 6G networks [22,23,24]. Forms of short-range technology, such as Wi-Fi and/or DSRC, are also used. Various types of sensors can be integrated into vehicles for engine, human health, and environmental monitoring. Sensors have become providers of multi-dimensional monitoring data. Sensors can also be used as information gathering sources, and thus collect contextual information, which can be related to the cargo transportation and management processes.

The integration of heterogeneous types of sensors can be included in the monitoring systems of the cargo transportation process. Our focus is on the following:

- Physical sensors for environment monitoring;
- Sensors for monitoring the working regimes of vehicles;
- Human health monitoring sensors.

The integration of sensors into the development of adapters for inclusion in smart service provision subsystems using the Geographical Positioning System (GPS) and geographical information system (GIS) has become popular. Sensors in the infrastructure of RSUs assist in the monitoring of transport and traffic information, provision of hazard warnings, recording interactions with other cars, e-calls for warning and information provision, etc. In the research of the development of advanced systems, the authors have offered several solutions for the development of adaptive service systems [3,7,8]. 

The problems related to gathering information from the environment and dissemination in dynamic environments are presented in [25]. Monitoring data must be linked to a higher level of representation via the level of ontology and other mechanisms of knowledge extraction. Questions regarding the understanding of the context have arisen in numerous research studies [18,21,26]. The definitions of context diversity and a short review of this analysis are presented in Table 1. Other authors have similarly defined the context, but with different interpretations. 

The concept of “context” is very broad. We analyzed these proposals of contextual data definitions according to the possibilities of integrating them into our system. The presentation of the broad spectrum of multi-dimensional aspects of contextual information with the intention of representing them in the smart service provision system allows the scope of recognition of this information to be extended.

V2V communication service provision is subject to a number of problems, i.e., the assessment of the quality of the gathered information and flooding of wireless channels [32,33,34,35]. A wide spectrum of communication protocols can be chosen in the architecture for gathering, transmitting, and dissemination of information. The requirements for IoT applications involve several aspects, such as adaptability, ingenuity, and flexibility [35]. However, we restricted our scope and did not analyze a variety of communication protocols. Regarding communication channels, our focus is on addressing the problem of flooding wireless channels caused by the provision of services. This approach concerns the evaluation of the priorities for service provision, with the purposes of ergonomics, safety for transportation, necessity, and adaptation. 

The concept of “Context Quality” (QoC) was established by [33] and can be interpreted as “*a set of parameters that reflects the quality requirements and properties of context data*” [34]. We are concerned with interdisciplinary concepts, and the interpretation of these concepts for the construction of AI systems, which have special abilities to combine the methods from behavioral science, operating and embedded systems, communication networks, and other fields. This creates a large gap between the high requirements for the creation of such systems, due to the complexity of working mobile systems, services, and operations, and the context that is derived from the environment. 

Due to the conditions of the heterogeneity of devices, a high level of mobility and changing topology requires the high quality of management of contextual information. Achieving this is extremely complex, and the errors and issues related to partial information can be significant.

Applications can take the contextual information from the context-aware modules and adapt their actions by following the changing context needs [36]. The management of contextual data, which are specified by the usage of a fast-changing topology under the conditions of the dynamically changing environment of transportation processes, and implementing the data from sensors that work under the background of vehicular communication networks, is challenging due to several factors [37]:

- *“Context becomes important due to the high mobility of nodes.*
- *Some kinds of context information are intricately linked with physical locations. But it may be challenging to update this information due to the repeating disconnection of nodes from the source of context.*
- *Relevance of temporary context is caused by dynamic changes and context gets temporary importance due to specifics of the dynamic transportation situations.”*

In services of V2V communication (in which ad-hoc networks can be created), the context may be ambiguous and redundant because, during the service provision process in ad-hoc networks, it is difficult to regulate the flow of contextual information. These problems were described in [38], in which the context state is analyzed as the collection of current sensor readings. 

The methods used to recognize contextual information have multi-attribute and multi-dimensionality properties. The multi-attribute utility theory (MAUT) was proposed by [39] for integration of heuristics rules and experts’ experience. Decision support systems commonly use the implementation of methods of weighting the parameters when it is possible to describe dynamic situations. Using the MAUT, the space of situations can be expressed in the model and, following the recognition of an adequate space, the information is provided by the model (e.g., the state of the context and the description of the expressed situation space). The model then calculates the degree of confidence with which the situation has occurred. The calculated reliability is compared with the confidence limit (a comparison of the calculated reliability with an individual situation of a certain threshold, which allows comparing the results calculated for different situations).

The dynamic properties of transportation are analyzed in many studies by revealing important properties of the contextual data [40]. Transportation and recording of information from dynamic objects involve heterogenic communication structures, and the importance of the context can differ in real-time updates. The situation assessment relates to the evaluation of the broader spectrum of given data, rather than that from an individual sensor. The background to this approach comprises the methods of recognition of context-aware data by classifying primary data sources that are obtained from different kinds of wireless sensors, such as speed gauges, accelerators, temperature gauges, radar positioning systems, and video provision systems.

## 3. An Approach for the Development of a Smart Service Provision System Applicable to the Management of Cargo Transportation Processes 

The objective of this research was the development of the extensible infrastructure of a smart service provision system for drivers in cargo transportation. The aim was to avoid accidents, assist in accident investigations, and/or assist in avoiding traffic congestion. 

An approach to the development of the adaptable smart service provision system is presented in Table 2. All layers are required for context-aware information recognition during the cargo transportation management process.

Investigations of the development of services related to vehicle-to-vehicle (V2V) and vehicle-to-infrastructure (V2I) communication are also important in our proposed approach. These processes provide decision support related to some of the levels of the hierarchy of decision making in cargo transportation management, with the aim of achieving the requirements of sustainable development.

Not all of these layers are described in this paper. However, we analyzed some of the layers in more detail as presented in the following sections.

### 3.1. Hierarchy of Decision Support Levels for Sustainable Management of Cargo Transportation

The decision support processes are introduced in the system using AI methods. These processes are related to the development of advanced services, which can help in the improvement of the whole management process during unsuspected and unwanted events. The stages of development of the smart service provision system are analyzed by including the transportation scheduling issues, and the recognition of the priorities of information transferred for drivers during the whole cycle of transportation.

Important characteristics of goods are recorded in CARNET-TIR cards (i.e., e-documents and their management system issued according to the Transport International Router and data recognition on TIR cards). These e-cards record information on the specifics of the goods and the transportation legislation.

It is assumed that in the planning stage, cargo transportation targets are set based on contractual agreements and projected delivery requests, and decisions are made in the planning phase. In the planning phase, the cargo transportation sequence is determined and transmitted to the control phase in the form of targets or defined points. In the collection phase, performance variables are included, ensuring tracking of the set values of infrastructure, driver, and vehicle. In the control and response phase, making decisions on conflicts is undertaken as:

- Leave the primary plan (“Leave plan”);
- Change the primary plan (“Change plan”);
- Remove the original plan (“Remove original plan”);
- Remove the incompatibility (“Remove incompatibility”).

Real-time measurements of process variables, and information on failures and disturbances, can be returned to the planning phase to inform transformation decisions.

The original plan can be scheduled due to some reasons and can be incompatible, i.e., there is equal likelihood for the original plan to be changed or canceled. After the decision is made, it is likely that it may be modified, by relocating with a new time. Alternatively, it may be left unchanged if there is no possibility to shorten or relocate the initial plan.

### 3.2. Requirements for System Development

To enable the development of a system that can organize transportation using safer, more reliable, more economical, and more comfortable vehicles, it is necessary to implement the achievements of many fields and, in particular, follow the requirements of, and possibly adopt, the DSRC (Dedicated Short-Range Communication) and IEEE 802.11p [41,42,43] as standards for Wireless Access in the Vehicle Environment (WAVE) [18,19,20]. These protocols allow a new level of service delivery to be achieved.

Services can be arranged in vehicles using the DSRC and the WAVE technology by allowing requirements of ITS development to be achieved, and by promoting road safety, more optimal traffic management, and provision of more comfortable services for drivers. 

The connection between the vehicle and the roadside units (RSUs) allows the necessary communication. In vehicles, it is possible to equip several types of onboard units (OBUs), and other units are dedicated external application units (AUs) [23,41,42]. The ad-hoc networking domain consists of vehicles that are equipped with an OBU. Vehicles can communicate with other vehicles by creating the structures of information, which change directly for the drivers, and comprise the MANET, which allows full distribution of information between vehicles via communication using precise coordination. RSUs can connect to the infrastructure using networks or Internet access points, thus providing the OBU with the ability to access the infrastructure of the network [19,20,23,41,42].

Attention has been paid to different types of possible transportation scenarios (in cities, across rural areas, crossing border points, uploading at terminals, etc.). In particular, the construction of identification algorithms for the recognition of different situations is needed to assess their importance for transportation management in an online regime. These methods are influenced by the service provision process for drivers. 

For analyzing warnings, we constructed algorithms that consist of the assessment of conditions of unpredictable situations for which warnings are needed (collisions, lane changing, etc.). To realize these algorithms, it is necessary to include the capability of emergency video transmission and other strategies for the prediction of events. These algorithms can help in the forecasting and re-planning of transportation circumstances, thus resulting in a system with greater predictive abilities for transportation management. The assessed information is disseminated, and the smart system is able to provide the information directly to drivers or receive it in the automatic active safety systems of DWs.

Services that provide comfort and entertainment are considered to be less important, and are thus not related to road safety. These types of services can be provided in normal driving situations and can help to improve the comfort of drivers (and/or passengers). Services provided at comfort areas can supply meteorological and traffic information, in addition to the details regarding the location and prices of the nearest petrol stations, hotels, etc.

The taxonomy of the VANET can be divided into a number of areas, such as field-level planning, control of the processes’ operational performance, and online access during the transportation processes. 

### 3.3. Architecture of an Intelligent Adaptable Service Delivery System for Cargo Transportation Process Management

The development of an intelligent adaptive service delivery system (IASDS) requires the integration of the immediate environmental identification and data management techniques. 

A more detailed conceptual architecture of the smart service provision system is proposed in our previous works [3,7,8]. Recently, special attention has been paid to the field of changing the topology of mobile networks and the development of algorithms for restricting the provision of unnecessary information. During transportation processes, drivers face an increasing flow of information. Special AI methods are integrated into the related systems to address the problem of obtaining useful knowledge from data sources, expert practices, and knowledge base (KB) structures. 

The question that arises in the development of a KB is how to adapt and construct a tailor-made level of service provision in real time, based on the knowledge gained from the context. In the provision of these services, a number of issues must be solved:

- The task of recognizing the necessity of the information that is linked with the response field, and assessment of its importance in developing the prioritization algorithm;
- The task of realizing automatic decisions about the services that will be provided based on the priority of the users (drivers or managers) in real-time processes;
- The task of expressing control models with mechanisms that enable the customization of service provision according to the user’s questions and behavior analysis;
- The task of identifying the security services that should be automatically provided, and recognition of their priority.

The raw data are recorded via acquisition processes using different types of sensors (Table 3).

**Figure 1:**
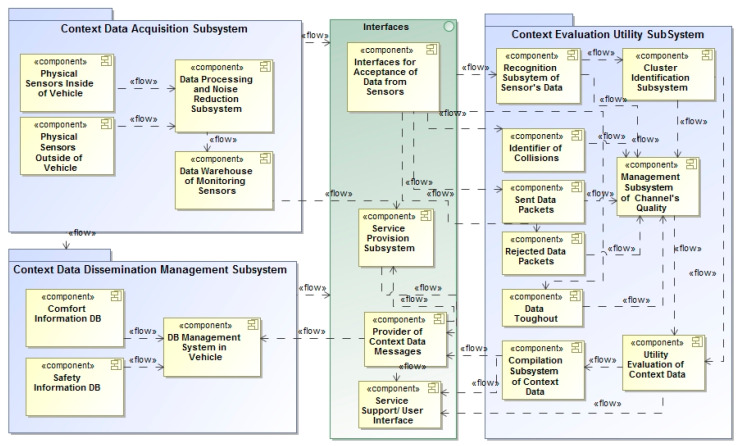
The architecture of the proposed IASDS for service support by recognition of data from sensors.

The main components of the structural architecture of the proposed IASDS are presented in Figure 1. For the representation of the component-based architecture of the system, we used the notation of the Unified Modeling Language (UML). 

Two main packages for data monitoring were created: the *Context data acquisition subsystem* (for integration of data monitoring functions obtained from the sensors) and the *Data dissemination subsystem*. The data from the acquisition subsystem are provided for evaluation modules, i.e., subsystems, which can provide context acquisition and dissemination functions of the contextual data. Working mechanisms are used for classification of services, development of prioritization of services, and acquisition of contextual information. A detailed description of these algorithms is presented in Section 5. The monitoring data are collected in data warehouses (DWs), which work in real time by applying cloud computing capabilities and gathering raw data from available sources.

The *Channel quality management subsystem* enables the performance and pre-processing of data to provide noise reduction. The interfaces play an important role as adapters for gathering data from different types of heterogeneous sensors, and can help in the integration of data into the conceptual structures of the repositories of the DWs. The data for processing are transmitted through the components of the interfaces, which are different to those of the embedded systems, from which data are transmitted for the *Context evaluation subsystem.* The algorithms of these systems can use the clustering method and formulate requirements for information flow in the *Situation identification subsystem*. 

The data for context acquisition are obtained from sensors (Table 2 and Table 4) and can be arranged locally and/or globally. Locally arranged sensors can provide information about the vehicle environment. Data from other vehicles have mixed characters. Data flows from sensors are classified and passed from the *Subsystems of service support* (Figure 1).

The KB includes the multi-component structure as follows:

- Computer-based ontology of the application domain of transportation;
- Rules of the management of transportation processes;
- Identification of important situations;
- Additional organization of decision support and control under the conditions of unexpected events.

Any identified situation implies that a certain mechanism should be used for the different transmission of information. The *Subsystem of situation identification* implies the provision of different services, which are selected using the capabilities of remote cloud service platforms. The subsystem of the service support interfaces is important, but we do not provide a detailed description of this component.

For a more detailed description of the types of communication under analysis, the infrastructure of communication between vehicles and RSUs can consist of three areas: the INV relates to the communication of the local area of the vehicle, which involves the OBUs and several application units (AUs). The ad-hoc net area consists of cars equipped with OBU devices and that can communicate directly with each other, thus forming the MANET, i.e., mobile ad-hoc network, which ensures fully distributed decentralized communication. The OBU devices can be classified according to WAVE, and are usually placed in the car’s local area and used for exchanging information between the RSU and other cars. The OBU has a *Resource Command Processor (RCP)*, which can explore the resources using read/write memory that is used for storing data in the DW. The OBU devices use interfaces that are dedicated to the type of communication that allows connection of the vehicle with network devices and other OBU devices using radio technology, by applying the IEEE 802.11p standard. 

Extra devices can implement other radio technologies, such as IEEE 802.11a/b/g/n/ac/ad. Mobile access protocols, such as 4G, 5G, and the upcoming 6G, can also be used, in addition to LTE, etc. The OBU devices can be connected to the RSUs or other OBUs via a wireless connection using the IEEE 802.11p channel for the radio frequency, which is responsible for communication with other OBU and RSU devices and provides communication services to the application device. The OBU provides the main functions of Wi-Fi access, ad-hoc and geo-location-based routing, network load management, reliable messaging, security, and IP mobility [21].

Using the AU device, the service provider realizes services based on the capabilities of the OBU. An AU can be a device dedicated to specific security programs or a regular device, such as a smartphone or tablet, that provides Internet services. The distinction between an OBU and an AU is often logical [41,42].

**Figure 2:**
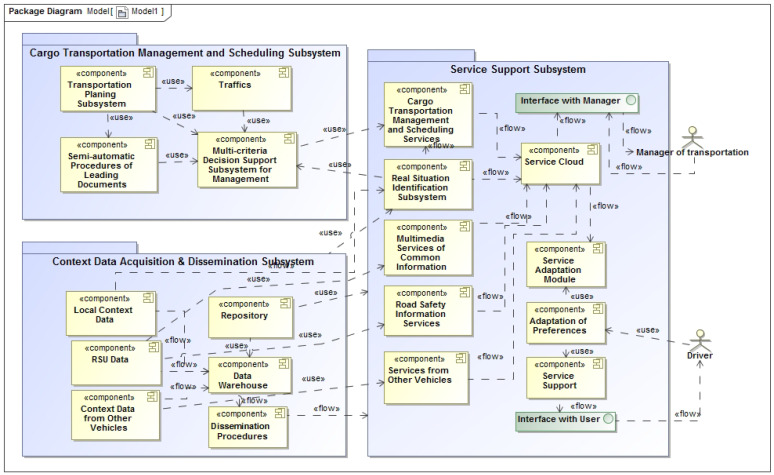
The architecture of the proposed IASDS for service delivery with the function of recognizing data from sensors.

The component-based architecture of the service delivery subsystem, and connection with other components and packages is presented, in Figure 2.

An RSU is the facility of WAVE devices that are installed near the road or in other specially designated areas, e.g., at intersections or parking lots. This device is equipped with short-range wireless technologies, such as IEEE 802.11p, or others that connect it to the network infrastructure. According to previous research [41,42], the main functions and procedures associated with RSU are as follows:

- Help to extend the distance of the ad-hoc network by forwarding information to other OBUs or RSUs;
- Help to run safety programs, such as crash warnings and road surface information, using the V2I mode and acting as a source of information;
- Provide OBUs for Internet access.

Areas of automotive communication:

- The INV domain consists of an OBU and one or more AUs. OBUs and AUs can be realized as a single device. In the automotive field, contextual information is collected from installed sensors and stored in a database [41,42].
- The V2V domain is the area of an ad-hoc type network that consists of cars equipped with an OBU. The cars communicate with each other via the OBUs, thus forming a MANET that provides communication in a fully distributed decentralized manner.

Cars can communicate directly with each other, if direct wireless communication between them is possible, by forming one-jump car-to-car communication (V2V). If direct communication is not possible, the data is transferred to other cars as intermediaries until the addressee is reached. These forms of multi-hop relationships are described in [21].

The infrastructure of the domain is related to all of these devices and, in particular, to the RSUs that connect to the infrastructure of the networks or the Internet by providing OBU access to that network. OBU devices communicate with various nodes by providing non-security services using other mobile technologies (GPRS, GSM, 4G, HSDPA, UMTS, and WiMAX) [41,42].

## 4. Conflict Resolution in the Actual Scheduling Process

The initial step is the identification of the conflicts that have arisen in creating the scheduling process. Operational incompatibility happens when a new unplanned situation occurs. It is important to note that there are cases that are not generally considered to be conflicts but as individual multifunctional operations. 

Once the existing conflicts in the data set have been collected, the response is necessary to help decide which strategies should be used to resolve them. To proceed, the revision step is used to determine whether a modification of the conflicting activity is in effect. For each conflicting couple, if one of the activities was undertaken, other modification activities determine the type of resolution. If neither activity was carried out, modifications of both activities are monitored to determine the possible resolution. Finally, when both activities are resolved, the incompatibility is deleted from the list of identified conflicts. In addition, to resolve the primary incompatibility of overlapping in time, all conflicts that were not caused by operational changes are eliminated from the list of conflicts and submitted for re-planning.

The user may not enter a large number of possible activities that conflict with the planned activity into the scheduler because these activities will be rejected directly and never planned. For this reason, the elimination of conflicting activities is not considered to be an option in the decision making, but is the default choice when any resolution strategy fails.

To create a *conflict resolution model*, the key attributes that should affect the conflict resolution process were first identified. The key attributes can be classified into the groups as follows [43]:

- Driver-related characteristics, including driving behavior and personal temperament. Driver-related characteristics reflect the socio-economic orientation of the vehicle’s driver, which is expected to affect cargo delivery needs and the ability to re-plan certain activities.
- Performance-related characteristics. These include the trip duration, vehicle location, fuel consumption, the aim of the trip, and the constructed route information. Performance characteristics define activities and, in general, reflect the amount needed and the degree of flexibility.
- Conflict-related characteristics. This group represents the type of conflict in which an overlap exists for the initially planned activity that is resolved by waiting until the activity is undertaken. It is considered that the symptoms listed above may have the greatest impact on driver expression behavior. The conflict-related characteristics determine the results of conflict resolution and priorities that are settled in repeating situations.

The main descriptive statistics on conflicts and decisions are used in the model’s construction. Compared to social and demographic variables, the signs of conflict play an important role in making decisions. When the number of overlapping initial activities increases, the number of deleted activities also increases and the number of modifications of activities decreases. However, the time available appears to have less impact on the decision selected. A small model reduces the likelihood of modification of the initial activity and increasingly deletes activities that appear to be in opposition. 

The specifics of the conflict activities were expected to have a significant impact on the resolution strategy chosen. In general, the original activity, which is characterized by greater flexibility, was likely to be only slightly more likely to be replaced. For example, original activities with more flexibility (leisure flexibility or driver personal flexibility only) have a higher modification percentage and a lower percentage of deletion. However, the results are not as clear as those in conflict activities. Regarding time horizon planning, it was generally observed that as the time horizon of the planning of both original and controversial activities increases (i.e., more are planned), activities are likely to be slightly changed.

The duration of the performance is the final characteristic of the activity. There is a noticeably clear pattern of gradually decreasing the deletion activities and increasing the modification of activities with increasing duration. For example, only one-third of short initial sessions (duration < 2 h) were modified compared to two-thirds of long sessions (>6 h); whereas one-third of short-term activities were deleted, compared with half of the long-term activities. This was expected because long-duration trips are also priority activities that cannot be easily carried over to the next day or missed. The tendency is less clear for conflict activities but is also much higher for the observed modification of exceptionally long conflict activities compared to deletion.

Frequency distributions of discriminatory strategies show some common patterns of interest, but interactions between variables and their relative importance cannot be determined in this manner. For resolutions of represented conflicts, some cases were analyzed. The model is expected to show similar trends as those noted above, but will capture the impact of each variable in more detail, in addition to the driver and time flexibility of the variables and their presence, which have not been considered before.

The scheduling of vehicles has been a subject of research for the past four decades. Many authors have investigated routing and planning aspects. Although many surveys have been conducted on the topic of vehicle planning, none of these consider the most recent research on modelling and decision methods. Because a large amount of new research has been undertaken in recent years, the authors provide an overview of the recent models. These models can be sub-divided into:

- Models that take into consideration relevant metrics, targets, or combinations of targets;
- Models that follow the integrative optimization of routes for further business decisions;
- Models that investigate more accurate and more pronounced aspects.

Most often, scheduling models consider costs as the main objective; however, these models overlook other important criteria, which are additional objectives. These criteria can be categorized into categories as follows:

- Performance: level of services, quality, penalty, navigation; congestions;
- Planning: work and drive balance, number of drivers, timing, duration of operations, number of uncertainties;
- Routing: locations, the quality of infrastructure, speed, type of freight, road taxes;
- Reliability: the probability of failure, truck readiness, driver behavior;
- Sustainable and environmental factors: fuel consumption, emissions, safety criteria.

The mentioned criteria may appear as the objectives (in a weighted, hierarchical, or multifunctional formulation) or as separate variables.

## 5. Description of Algorithms for Recognition of Priorities of Contextual Data Provision during Cargo Transportation Processes

For the cargo transportation process, it is necessary to build the services with adaptation properties. The intelligent adaptable smart service delivery system (IASDS) under development can be constructed for the autonomous provision of these services for drivers. Algorithms must be constructed to define the priority of service provision when it is at the right time and the right place. The system is aware of the surrounding environment and can help to identify the current, past, and likely future situations throughout the trip’s duration, and help to choose the right direction. 

The infrastructure of road equipment is supported with a variety of sensors. The information (data) is provided from a wide spectrum RSU and can help in monitoring transport and other types of contextual data throughout the journey. Sensor data can be used for situational awareness. These monitoring data produced during the transportation process are highly complex. The data structures can have different provision modalities, different measurements can have significant volumes, and different kinds of complex interdependencies can exist between sources. 

The key factor in the IASDS development is the identification of situations via the understanding of relationships between them and their context, and the management of these situations. If this is not achieved, the system may function incorrectly and not properly adapt to the needs of the user. The system should be aware of several simultaneous situations or the fact that they cannot occur simultaneously, e.g., a car cannot be parked and driven on a highway at the same time. It is challenging to simultaneously address the complex operating conditions of the system, the high level of dynamics, the heterogeneity of sensors, the inaccuracy of sensors, and other circumstances. The situation is influenced by vehicle movement scenarios. Differentiation appears if transport is classified by highway or may be inherent to the scenario.

From analysis of the research subject, the following questions arise:

- How can contextual information be obtained?
- How can this information be disseminated in vehicle networks?

We use the definition of the term “contextual information” as “any information that can be used to describe an entity’s situation, whether the entity is a person, place or object the interface between the user and the system, including the same user and system” [31].

The component of the system that rejects non-useful information can help in reducing network loads, but without losing data quality or reducing the efficiency of road safety in the multimodal system. To address these issues, an intelligent adaptive approach was proposed that allows the use of environmental contextual information, including location, time, environment, user status, vehicle dynamics, and information from other cars, while considering network conditions and the available resources for data packet formation and transmission solutions. 

Considering the identified environmental parameters, it was proposed to create a subsystem of the context assessment (based on a software system) that evaluates the usefulness of each contextual message. The data warehouse (DW) of the sensor data is extended by a meta-model, and is constructed for local storage by implementation of the mathematical background. An algorithm structure was formed that can provide a more formal description of the data transmission procedures that must initiate the transfer of information to other nodes, and/or the transmission procedures to the server.

The matrix *M_L_* is constructed for evaluation of the usefulness of each data message, which can be expressed as a Cartesian product. The values express the usefulness of messages of local context, where the first index *i* of *d_Lij_* expresses the data form message obtained from the sensor (*s_j_*), for the index *j*:

(1) $$M_{L}=\left(\begin{array}{cccc} d_{L_{11}} & d_{L_{12}} & \ldots & d_{L_{1n}}\\ d_{L_{21}} & d_{L_{22}} & \ldots & d_{L_{2n}}\\ \ldots & \ldots & \ldots & \ldots \\ d_{L_{l1}} & d_{L_{l2}} & \ldots & d_{L_{ln}} \end{array}\right)$$

The predictive usefulness of the specific contextual data message is weighted by a function that assigns values to each data message (*m*_i_) from sensors (*s_j_*). The values of items of matrix *M_L_* are calculated according to Formula (2):

(2) $$d_{L_{ij}}=\left(Ty_{j}+H_{j}+Ex_{j}\right)m_{i}cr_{i}Pr_{i},\;i=1,\ldots ,l,\;j=1,\ldots ,n\;$$

In Formula (2), *Ty_j_* express the values that express the importance of the contextual data types, which are assessed within the range of [1, 3]. These assignments are formatted by the opinions of specialist experts in the transport sector. For the formulation of these assessment algorithms, we conducted surveys and applied multi-criteria evaluation methods for the comparison of opinions of specialist experts. A value of 1 is assigned for expression of messages from comfort areas; 2 is assigned to messages with a mixture of comfort and safety areas; and 3 is assigned to messages from the security area. The messages with a weight of 3 are provided first.

*H_j_* is the set of parameters that indicate the importance of the message, including its importance for future decisions. The values are assigned as follows:

- When the message should be stored for a longer time, and its value will be used for historical assessment needs, the assignment of the message weight is equal to 1;
- If the message is not important for future decisions, it is assigned a weight of 0.

The parameter *Ex_j_* expresses the data exchange area and digital values are assigned within the range [1, 4] as follows: 1—when the message is from *Vehicle to Manager (V2M)* communication; 2—when the message is in the local environment of the vehicle (*INV*); 3—when the message is from *Vehicle to Infrastructure (V2I)* communication; 4—when the message is from *Vehicle to Vehicle (V2V)*. In addition, a set of parameters *Cr_j_* expresses the coordinates at which the data message was generated at the real-time point *t_i_*; these values are expressed as *cr_j_^t^_i_ =* {*cr_j_^t^_i_^,latitude^; cr_j_^t^_i_^,longitude^*}.

The message priority can be calculated by the formula ${Pr}_{j}=1+\frac{I_{j}}{A_{j}}$, and values are assigned according to the normalized values of the set [1–3]:

- If the assignment for the message is *Pr_i_* : = 3, it means that the message *m^tj^_i_* priority at the moment *^tj^* is critical and it must be sent immediately to the driver and stored in the corresponding DW;
- If the assignment for the message is *Pr_i_* : = 2, it means that the message *m^tj^_i_* at the moment *^tj^* has a medium priority;
- If the assignment of the message is *Pr_i_* : = 1, it means that the message *m^tj^_i_* at the moment *^tj^* is not important and can be rejected.

The importance of the message can be assigned within the set range [0, 1], where 1 indicates a safety-related message and 0 indicates a comfort/information message. 

$I_{j}$ is the function for expression of the message age with normalized values, which can be chosen from the set of values from within the interval [1, 3], which is calculated according to the algorithm that is expressed in (3), where *T_M_* is the difference between the current time moment and the time moment when the message was created.

(3) $$A=\{\begin{array}{c} {\;1,\;\mathrm{if}\;T}_{M}>5s\\ 2,\;\mathrm{if}\;1<T_{M}<5s\\ {\;3\;\mathrm{if}\;T}_{M}<1s \end{array}$$

To reduce the number of data transitions, the structure and algorithms were developed to help achieve better conditions in the provision of selected messages. The matrix *M_O_* is used for storing the context utility values. The items *d_oln_* of the matrix *M_O_* indicate the importance of the data, where the *l* index indicates the data from a message (*m*) provided for the vehicle *n* (*v*) (4), where *v* represents vehicle.

(4) $$M_{O}=\left(\begin{array}{cccc} d_{o_{11}} & d_{o_{12}} & \ldots & d_{o_{1n}}\\ d_{o_{21}} & d_{o_{22}} & \ldots & d_{o_{2n}}\\ \ldots & \ldots & \ldots & \ldots \\ d_{o_{l1}} & d_{o_{l2}} & \ldots & d_{o_{ln}} \end{array}\right)$$

The predictive usefulness of a contextual data message is weighted by a function that assigns a value $m_{l}$ to each message to be transmitted to the vehicle (car) $v_{n}$. The values of the items of the matrix *M_O_* are calculated according to Formula (5), which can be expressed as the Cartesian product:

(5) $$d_{o_{ij}}=\left(Ty_{j}+Exc_{j}+Z_{j}\right)m_{i}{cr}_{i}Pr_{i}n_{i},\;i=1,\ldots ,l,\;j=1,\ldots ,n$$

where *Exc_j_* is a parameter of a special non-confidential data set, for which assignments are chosen from the range [1, 4], indicating the data exchange area (1—V2M, 2—INV, 3—V2I, 4—V2V); *n* indicates the number of cooperating cars in the cluster; and *Z_j_* is the communication channel quality prediction indicator that can be calculated based on Formula (6):

(6) $$Z_{t}=\frac{1+\left(\frac{C_{t}+D_{t}}{2}\right)}{Tr}$$

where *C_t_* is the collision parameter calculated by the formula: $C=1-\left(\frac{1}{1+c_{t-1}}\right)$; 

*D_t_* is a packet of rejection parameters, which can be calculated according to $D=1-\left(\frac{1}{1+d_{t-1}}\right)$; and *Tr* is the bandwidth parameter calculated according to the formula: $Tr=1+\left(\frac{{tr}_{t-1}}{100}\right)$.

The contextual data for exchange with a hybrid VANET cloud subsystem contain the context utility values. These are stored in the structure by representing the matrix (*M_c_*); their index *l* expresses the values of the data of the messages (*m_j_*) for the recipient entities (*r_i_*) by presenting such a matrix as (7):

(7) $$M_{C}=\left(\begin{array}{cccc} d_{C_{11}} & d_{C_{12}} & \ldots & d_{C_{1n}}\\ d_{C_{21}} & d_{C_{22}} & \ldots & d_{C_{2n}}\\ \ldots & \ldots & \ldots & \ldots \\ d_{C_{l1}} & d_{C_{l2}} & \ldots & d_{C_{ln}} \end{array}\;\right)$$

The values of the usefulness of the contextual data messages are predicted and can be expressed by the weighting function, which assigns the values to each message that is to be passed to the recipient entity. The values of this structure can be calculated according to Formula (8) expressed as the Cartesian product:

(8) $$d_{C_{ij}}=\left(Ty_{j}+Hx_{j}+Exc_{j}+Z_{j}\right)m_{i}{cr}_{i}Pr_{i},\;i=1,\ldots ,l,\;j=1,\ldots ,n\;$$

where *Ty_j_* is the set of reduced parameters. *Ty_j_* is assigned values from the range [1, 2], where value 1 is assigned to messages from the comfort area and value 2 is assigned to messages from the comfort and safety area. *Hx_j_* is a parameter for a special non-confidential data set from which values are assigned within the range [0, 1], by indicating whether the data should be stored historically.

## 6. Experimental Research Possibilities with the NCTUns Simulation Tool for Testing the Algorithms of IASDS

The prototype of IASDS was tested in a series of experiments using simulation methods and packages for these purposes. One of these allowed the analysis of the adaptability of the data aggregation via an evaluation of the adaptability of the provided algorithms. The algorithms (described in Section 5) were simulated using the National Chiao Tung University Network Simulator (NCTUns) modelling package, following the recommendations presented in [44,45]. The NCTUns package enables the high-precision expression of the extensible integrated network and mobility. In addition, it includes a modelling tool and an emulator. NCTUns allows the modeling of a wide variety of protocols that are used in both wired and wireless networks. A modern kernel re-entering method allows different experiments to be undertaken with NCTUns. 

An intuitive user interface was used that eliminated the need for complex scriptwriting. The benefit of NCTUns and its later version (Estinet) [44] is that they can support the basic patterns of a driver’s behavior. In particular, ITS researchers have focused on the functions developed in NCTUns versions 4.0 and 5.0, which allow experiments with ITS automotive networks, basic road network design, RSU modelling, and the imitation of OBUs that can be equipped with wireless IEEE 802.11 (b) infrastructure mode. In addition, the ad-hoc mode, GPRS, 802.16 (e) modelling of mobile WiMAX communication technologies, and DVB-RCST satellite communication was introduced. During the experiments, a wide spectrum of possible wireless access methods were applied. Because this modelling environment is tightly integrated, it can be used to investigate sets of complex ITS situations. This is required in the simulation of changes in the car’s driving behavior after receiving a certain set of messages from the network. NCTUns version 5.0 introduced important enhancements to VANET network modelling, namely, efficient node mobility management, ultra-high volume automotive communication networks, automatic road network construction, and, most importantly, the IEEE 802.11 (p)/1609 standard for automotive communication network support. 

According to the purposes of the experimental research, a number of scenarios of the simulation process were carried out. The aim of one scenario was to simulate the network model for the expression of processes that created a series of messages in which data were transmitted from the vehicles to the hybrid VANET cloud server, by storing appropriate information in adequate structures of the repositories of the data warehouse (DW). The network was related with the interoperable server and the DW. A number of 802.11p RSU units were implemented in the simulation laboratory and, during this scenario, we simulated from one to ten 802.11p OBUs. 

For this scenario, we obtained the statistical data relating to the parameters of transmission, the technical parameters of messages (Table 4), and other statistical data [45].

To complete the algorithms (Section 5) used to assign priorities for the different types of context and provided messages, the opinions of specialist experts from the transport sector were analysed. To assign concrete priority values in the provision of contextual information and services, we used multi-objective decision support methods, such as additive waiting and evaluation of concordance coefficients, for the assignment of the priorities of the experts. 

Another simulation scenario was used to analyze the data flows by transferring them in two directions: from the vehicle to the DW server and from the DW server to the vehicle. The types of sensors applied in this experimental scenario are presented in Table 5.

The duration of the data transfer cycles started at 60 s. The data transfer speed was chosen to be 27 Mb per second and the size of the packet was approximately 1000 MB. The experiment included the algorithm for defining the priority assignments of service providers described in Section 5. The prototype system was simulated with the functionality of context-awareness of the surrounding environment. 

**Figure 3:**
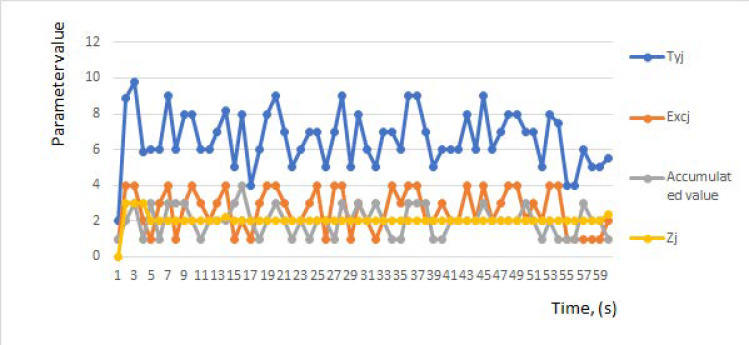
Illustration of the partial experimental results representing the dynamics and dependency between changing values of estimated variations of the accumulated predicted utility (*Exc_j_*), normalized *Z_j_*, and *Ty_j_* parameters during a short period.

Another group of experiments was undertaken with the aim of identifying the potential for application of the algorithms provided in Section 5. Statistical data and collected data were used during short intervals. For illustrative purposes only, the trends over time (in seconds) of parameters, namely, the accumulated values, the dynamics of the values of predicted utility *Exc_j_*, and the dynamics of the obtained values of normalized function *Z_j_*, are shown in Figure 3. The dynamics of the reduced importance of the *Ty_j_* values are shown in the same figure for comparison purposes. 

These results are not accurate and only have demonstrative ability. Nonetheless, they show the possibility of simulating the performance of changes in the service provision process by assessing the accumulated values over the short experimental period. The changes in the values depend on the parameters of the channel quality, among others. To include the estimation of situations involving collisions, and the required bandwidth of the channels, it is necessary to carry out additional experiments with a number of dropped packets. 

The prediction function can help to identify the current situation by evaluating past events, and be used to potentially predict the future situation, at the stages throughout the duration of the trip. The algorithms for selecting the correct direction at possible points (nodes) of multi-modal transportation were presented in our previous work [47]. 

## 7. Discussion

The processes of cargo transportation must be equipped with new ICTs and methods of communication using wireless sensor networks (WSNs), by implementing the platform of IoT technology. The development of adaptable smart service provision systems considered the complexity of these processes. 

The authors devised an approach for the integration of heterogenic sources of monitoring data during cargo transportation processes, aimed at the provision of the necessary information by analyzing the methods of context-aware information recognition and scheduling. Algorithms were developed that express and apply the methodology of these methods. A review of related research was undertaken to help provide an understanding of the manner in which this data can be applied for context-aware information recognition. 

It is intended that the presented approach can be used to develop an adaptable system for cargo transportation, with extended properties for the integration of context-aware services. The prioritization methods are included in the algorithms of service provision, which enable the restriction of unnecessary information. The architectural structure of the smart service provision system was developed based on WSNs and communication channels, thus enabling an improvement in the creation of delivery systems of smart service provision. The development of adaptable smart service provision systems considered the complexity of such processes. The online decision-making processes involve the assessment of all infrastructure components that are required for monitoring cargo transportation, and ensure the implementation of the necessary equipment. 

Our future research investigations will address the more complex scenarios of accident event recognition and the prediction of unexpected situations. In the future, the classification of infrastructure components according to different types of wireless technology, and the transfer of context-oriented data for specific smart service designs, will be implemented to reduce the number of accidents and unsafe situations.

## 8. Conclusions

The described approach is based on the extensible infrastructure of the required components and multi-dimensional methods for knowledge representation. The aim was the development of an intelligent smart system for service provision with the ability to recognize context-aware information. The authors integrated a wide spectrum of contextual data sources into the system development process; these data can be gathered from WSNs and other infrastructural ICT devices. The review of the literature highlights the investigations into these context-aware processes, the systems development required to realize the important context-aware recognition purposes, and the influences on the broader understanding of contextual information. 

The proposed structural architecture of the intelligent service delivery systems is unique due to the algorithms created for the identification of situations via the understanding of their context. In addition, the presented approach formulated a basis for the development of adaptable software methods, which are key to the automation of the management of these situations. To address the challenges related to smart service provision, the proposed intelligent adaptive approach allows the use of environmental contextual information, including the vehicle dynamics, and information transmission from other vehicles. By considering the wireless network conditions and the evaluations of the effectiveness of wireless communication channels, data transmission restriction methods were applied. These methods aim to assist the service restriction process by avoiding possible unexpected situations according to the capacities of the wireless communication channels. 

The proposed IASDS prototype implemented the automotive communication network infrastructure. The IASDS can help reduce the amount of unusable contextual information, which must be transmitted in automotive communication networks, without the loss of contextual data quality, using network filtering and aggregation techniques. The authors provided the classification of sensors by type, which was applied to the recognition of specific situations. The results of this approach identified the effectiveness of the methods that were developed to collect data.

The study has some limitations that could be researched in future studies. The typology of unplanned events was not presented. The authors would also like to investigate the number of road accidents that involve heavy vehicles. Other potential research topics include tire damage, unexpected vehicle repair needs, policy stops, traffic jams at customs offices, and route mistakes.

The proposed smart service provision system will be helpful in the analysis of the heterogeneity of services, the construction of algorithms for service provision priorities, and the enhancement of the potential of intelligent transport, based on the recognition capabilities of context-aware information in the transportation process.

## Figures and Tables

**Table 1:** Review of the analyzed aspects of the contextual information related to transportation.

The Domain of Analysis of the Context	Definition and Analyzed Aspects of the Context in Transportation	Works of Authors
Surroundings and people factors	Location and identity of surroundings, people, and objects, and changing objects	[26]
Localization and meteorological data recognition	Location and identity of people around the user, time of day, time of year, temperature, etc.	[27]
Conceptual definition	Set of entities that interact physically with conceptual states	[28]
Application factors	Application settings	[29]
Environmental surroundings	Environment reification, or anything that describes the surrounding environment in which the system performs	[30]
Environmental factors	Time, environment, product, and company information	[31]
Circumstantial factors	A set of circumstances or facts that surround a particular event or situation	[32]

**Table 2:** Approach for the representation of layers of the adaptable smart service provision system based on context-aware information of cargo transportation processes.

The Layers of the Infrastructure for the Development of Context-Aware Services	Description of Components and Functional Units
Software and algorithms for service support and specialized user interfaces	Software development tools for such purposes; Programming languages; Service provision platforms; Interoperability support tools for integration of required DWs.
Scenarios and orchestration of activities for service provision	
AI methods for recognition of cargo transportation situations	Special AI methods that are included in service provision systems, such as machine learning methods and packages, neural networks, and image analysis and recognition.
Methods for planning and/or re-planning of cargo transportation cycles	Plans to develop software, planning of transportation corridors, provision of related information, re-planning decisions and related activities, software realization tools.
Methods for operative work and management of all participating agents in cargo transportation processes	Operative control methods and implementation of informational and equipment infrastructure for communication between participating sides.
Rules and knowledge base (KB) for interpretation of obtained context-aware information	Spectral definition of contextual information, classification methods, multi-criteria decision support methods, production rules, fuzzy logic inference mechanisms, etc.
Computer-based ontology, conceptual models, and meta-model structures of DWs of contextual data	Conceptual models of integrated DBs and DWs; Languages for computer-based ontology development (e.g., Ontolingua, Protege, UML); Metamodels and conceptual structures of repositories for managing big data DWs.
Data warehouses (DWs) for monitoring all types of information required for the cargo transportation process	Implementation of cloud computing and technology platforms of IoT for moving objects; Communication networking tools; Interoperability support platforms for DWs.
ICT support for context-aware information gathering and obtaining	Techniques and equipment of road networks; Wireless communication technologies, WSNs, GPS, GPRS, satellite communication; Online GIS; Infrastructure support for communication with roadside units (RSUs), onboard units (OBUs), application units (AUs), DSRC, etc. Equipment for user interfaces (UI); Ad-hoc communication means, V2V, V2I, and all necessary communication standards, such as IEEE 802, 11 (p)/1609.
The infrastructure of networks of transportation roads and corridors including all underground means of transportation	The network of roads, terminals, reloading points, cross-border points, goods stores, and means of transport; DBs and DWs for the description of networks of roads of transport corridors and all physical means; E-document management systems; CARNET-TIR international systems; Sensors and monitoring systems related to the management of cargo transportation; Hydro-meteorological information systems; Operative IS for road network conditions, plans of road repairs, delays, and partial destruction; Traffic information for detection of abnormal situations.

**Table 3:** Types of sensors that can be equipped in means of transport and RSUs for contextual data assessment.

Types of Sensors That Can Be Equipped in Vehicles (INV)	Types of Virtual Equipment	Types of Communication Network between Vehicles	Types of Roadside Units (RSUs)
Video cameras	Smartphone/Tablet	VANET	RSU for speed recognition
GPS	Calendar	Services provided from ad-hoc nets	Dynamic RSU for traffic regulation
Microphones	Reminder	Reminders	Dynamic info black-boards
Movement dynamics	Information from social networks	V2V communication services	Monitoring and information about conditions of roads
Sensors providing vehicle engine working parameters and fuel assumptions	CARNET/TIR mark equipment	WiMAX communication	Sensors for meteorological conditions
Power hit sensors	Documents about planned activities, changing of time scheduling, and primary plans	Wireless IEEE 802.11 (p), and 12 and higher infrastructures	Information regarding road directions, official repair works, etc.
Temperature measurements (inside and outside of means of transport)		DVB-RCST satellite communication and other possible wireless methods	

**Table 4:** The assessment of the data transfer parameters needed for the experiment on the heterogeneous service provision in vehicular communication networks.

Activity and/or Service Type	Required Amount of Transferring Package (B/s)/Bandwidth (KB/s)	Possibility of Influence of Packet Loss	Frequency of Transmitted Data	Tolerable Delay (in Microseconds)
Types of actions related to traffic safety in the transportation process				
Changing the lane	~100/1	Average	Event	~100
Information for traffic light control system	~100/1	Average	Periodical	~100
Warnings about hazards	~100/1	High	Event	~100
Multimedia services	~100/1	Average	Periodical	~100
Multimedia services from comfort area				
IPTV	~1300/500	Average	Periodical	<200
VOIP	~100/64	Average	Periodical	<150
Exchange of video/audio packages	Higher	High	Periodical	~200 and interruptions, if transmission is possible
Games	Higher	High	Periodical	~300, if transmission is possible -

Source: data were prepared according to previous research [46].

**Table 5:** Classification of sensors, by type, that were applied for the recognition of contextual data from WSNs in cargo transportation.

Classification of Sensors by Types of Applicability	Update Rate (High—H, Average—A, Low—L)	Classification of Types of Row Data Sources	Types of Data Exchange
Physical sensors inside of the vehicle			
Speed measurement	H	Data from vehicles	INV
Acceleration measurement	H	Data from vehicles	INV
Temperature measurement inside the vehicle	L	Data from vehicles	INV
Temperature measurement outside the vehicle	In case of needfulness of such conditions	Data from vehicles and RSU	INV
Fuel level measurement	L	Data from vehicles	INV
The number of passengers measurement	L	Data from vehicles	INV
Vision	H	Data from vehicles	INV
Transmission of voice given commands	Average	Vehicles	INV
Millimetre-wave radar system	H	Data from vehicles	INV
Physical sensors outside of the vehicle			
Global location position (GPS)	H	Data from vehicles	INV, V2I
Quality of Wireless Sensor Networks (WSN)	A	Data from environment	V2I
Information about wireless interface	L	Wireless interface equipment	INV, V2I
Virtual additional types of equipment			
Calls	L	Data from smartphones	V2M
Calendar information	L	Data from smartphones	V2M
Notification of reminders	L	Data from smartphones	V2M
User preferences	L	Data from smartphones	V2M
Information about road	H	Data from vehicles, governmental institutions, and the environment	V2I, V2M, V2V
Notification of warnings	H	Data from another vehicle, governmental institutions, and the environment	V2I, V2M, V2V
Information about V2V communication	A	Data from environment	V2I, V2M, V2V

Source: data were prepared by following the research results in [8,19,23,24].
